# Bowman‒Birk Inhibitor Suppresses Herpes Simplex Virus Type 2 Infection of Human Cervical Epithelial Cells

**DOI:** 10.3390/v10100557

**Published:** 2018-10-12

**Authors:** Yu Liu, Xi-Qiu Xu, Biao Zhang, Jun Gu, Feng-Zhen Meng, Hang Liu, Li Zhou, Xu Wang, Wei Hou, Wen-Zhe Ho

**Affiliations:** 1 School of Basic Medical Sciences, Wuhan University, Wuhan 430071, China; liuyu2016@whu.edu.cn (Y.L.); xiqiu0810@whu.edu.cn (X.-Q.X.); zhangb1004@whu.edu.cn (B.Z.); gujun@whu.edu.cn (J.G.); fengzhenMeng@whu.edu.cn (F.-Z.M.); daisy_dec@whu.edu.cn (H.L.); zhouli_jerry@whu.edu.cn (L.Z.); houwei@whu.edu.cn (W.H.); 2 Department of Pathology and Laboratory Medicine, Temple University School of Medicine, Philadelphia, PA 19140, USA; xuwang@temple.edu

**Keywords:** Bowman‒Birk inhibitor (BBI), herpes simplex virus type 2 (HSV-2), antiviral activity, NF-κB, ubiquitin‒proteasome system (UPS), antiviral ISGs

## Abstract

The Bowman‒Birk inhibitor (BBI), a protease inhibitor derived from soybeans, has been extensively studied in anti-tumor and anti-inflammation research. We recently reported that BBI has an anti-HIV-1 property in primary human macrophages. Because HSV-2 infection plays a role in facilitating HIV-1 sexual transmission, we thus examined whether BBI has the ability to inhibit HSV-2 infection. We demonstrated that BBI could potently inhibit HSV-2 replication in human cervical epithelial cells (End1/E6E7). This BBI-mediated HSV-2 inhibition was partially through blocking HSV-2-mediated activation of NF-κB and p38 MAPK pathways. In addition, BBI could activate the JAK/STAT pathway and enhance the expression of several antiviral interferon-stimulated genes (ISGs). Furthermore, BBI treatment of End1/E6E7 cells upregulated the expression of tight junction proteins and reduced HSV-2-mediated cellular ubiquitinated proteins’ degradation through suppressing the ubiquitin‒proteasome system. These observations indicate that BBI may have therapeutic potential for the prevention and treatment of HSV-2 infections.

## 1. Introduction

Herpes simplex virus type 2 (HSV-2) is an enveloped double-stranded DNA virus that belongs to the herpesviridae family. Due to its highly prevalent and contagious trait, HSV-2 has been considered as one of the most severe pathogens for human beings. HSV-2 infection can cause genital herpes, which plays a significant role in the spread of sexually transmitted infections (STIs). In addition, through mother-to-child transmission, HSV-2 is the cause of neonatal herpes, responsible for high morbidity and mortality [1]. The latest report from WHO showed that the global overall rate of neonatal herpes was estimated to be about 14,000 cases (10,000 for HSV-2; 4000 for HSV-1) per year [2]. More importantly, there is increasing evidence that HSV-2 is a risk factor for HIV infection and sexual transmission [3,4,5]. The coinfection with HSV-2 is associated with a severity of genital ulceration, a reduced HIV-specific T cell response, and an increased systemic inflammation [6].

Human cervical epithelial cells in the female reproductive tract (FRT) are the primary barrier for protecting the host from pathogen invasion, as they have a significant role in FRT mucosal innate immunity against viral infections, including HSV-2 [7,8]. Studies have documented that the activated epithelial cells could produce specific cytokines, antiviral factors, and adhesion molecules to inhibit viral infections [9,10]. Our early work demonstrated that human cervical epithelial cells possess functional toll-like receptor 3 (TLR3) signaling pathway, which can be immunologically activated to protect genital mucosa from HSV-2 infection [11], and to inhibit HIV replication in human macrophages as well [12]. As the first layer cells in FRT, cervical epithelial cells are in direct contact with invading viruses. Therefore, understanding the processes and mechanisms that protect cervical epithelial surfaces from viral infections is clinically relevant and significant.

Clinically, several nucleoside analogs, such as acyclovir (ACV) and penciclovir (PCV), have been used to treat HSV infection as they can inhibit HSV DNA replication and reduce the frequency of ulcerations [13,14]. However, the emergence of drug resistance has become a problem for the treatment of HSV-2, especially in immunocompromised subjects [15,16,17]. Therefore, the search for new anti-HSV compounds, particularly those derived from natural products, remains if great interest in the field. It has been reported that elafin (E), human serine protease inhibitor, and its precursor, trappin-2 (Tr), can directly act on epithelial cells to reduce viral attachment and decrease NF-κB nuclear translocation, resulting in inhibition of HSV-2 infection in female genital mucosa [7]. BBI is a natural serine protease inhibitor extracted from soy, which is a monomeric protein with the inhibitory activity of trypsin and chymotrypsin [18]. BBI is present in many commercial soy foods, including soymilk, tofu, and soybean-based infant formula. BBI has been reported to possess anti-tumor [19,20], anti-inflammation [21,22,23], and antiviral activities [24]. We recently documented that BBI has the ability to induce intracellular antiviral factors that inhibit HIV-1 replication in human macrophages [25,26]. Because HSV-2 infection is a cofactor promoting HIV-1 sexual transmission, we examined whether BBI has an inhibitory effect on HSV-2 infection of human cervical epithelial cells. In addition, we investigated the mechanisms for the effect of BBI on HSV-2 infection.

## 2. Materials and Methods

### 2.1. Cell Lines and Virus

Human End1/E6E7 cell line is derived from normal human cervical epithelium, which is immortalized by human papillomavirus type 16 (HPV 16) E6/E7 [27]. The cells were cultured in keratinocyte growth medium (Gibco, Grand Island, NY, USA) containing bovine pituitary extract (50 μg/mL) and recombinant epidermal growth factor (0.1 ng/mL). African green monkey kidney epithelial cells (Vero) were cultured in Dulbecco’s modified Eagle’s culture medium (DMEM, Gibco, Gran Island, NY, USA) supplemented with 10% fetal bovine serum (FBS, Gibco) at 37 °C in a humidified atmosphere of 5% CO_2_. HSV-2 G strain was kindly provided by Dr. Qinxue Hu (State Key Laboratory of Virology, Wuhan Institute of Virology, Chinese Academy of Sciences, China). The HSV-2 G strain was propagated at a low multiplicity of infection (MOI) in Vero cells.

### 2.2. BBI

Bowman‒Birk inhibitor (BBI) was purchased from Sigma-Aldrich (St. Louis, MO, USA) (CAS #: 37330-34-0). BBI powder was dissolved in sterile double distilled water (10 mg/mL) and stored at −80 °C.

### 2.3. Reagents

Antibodies against NF-κB p65, phospho-NF-κB p65 (Ser536), p38 MAPK, phospho-p38 MAPK (Thr180/Tyr182), STAT3, phospho-STAT3 (Tyr705), STAT1, phospho-STAT1 (Tyr701), IRF3, IRF7, MxA, ISG56, OAS1, ubiquitin, and ZO-1 were obtained from Cell Signaling Technology (Danvers, MA, USA). Antibodies against p53, ISGF-3γp48, Occludin, and Claudin-5 were purchased from Santa Cruz (Dallas, TX, USA). Antibodies against HSV1+HSV2 gD, and OAS2 were purchased from Abcam (Cambridge, UK). Antibody against GAPDH was purchased from Proteintech (Chicago, IL, USA).

### 2.4. Cell Viability Assay

The cytotoxic effect of BBI was evaluated by the MTT assay according to the manufacturer’s instructions (Sigma-Aldrich). End1/E6E7 cells (10^4^ cells/well) were placed in 96-well plates and treated with different concentrations (100, 200, 400, 600 μg/mL) of BBI for 96 h. Cells were exposed to MTT (100 μL/well) and incubated for 4 h at 37 °C in darkness. Absorbance at 490 nm was measured by a plate reader (SpectraMax i3, Molecular Devices, Sunnyvale, CA, USA).

### 2.5. In Vitro Antiviral Assay

End1/E6E7 cells were pretreated with BBI for 24 h and then infected with HSV-2 (MOI of 0.001) for 2 h. The cells were washed three times with PBS to remove unattached viruses and cultured with or without BBI for 48 h. HSV-2 genome DNA from HSV-2-infected cells or culture supernatant was extracted with DNA lysis buffer as previously described [11]. HSV-2 gD copies were used to measure the degree of HSV-2 replication, which was quantified by the real-time PCR using the HSV-2 gD-specific primers (Table 1). HSV-2 gD standards with known copy numbers were used to quantify HSV-2 gD copies in the culture supernatant. In addition, the antiviral effect of BBI under different treatment conditions (before, simultaneously, after, and all) was evaluated. Briefly, End1/E6E7 cells were pretreated with BBI (200 μg/mL) for 24 h, then washed with PBS, infected with HSV-2 and then cultured without BBI (before); End1/E6E7 cells were simultaneously (simul) treated with BBI and infected with HSV-2 for 2 h, then washed to remove unattached viruses and cultured without BBI; cells were first infected with HSV-2 for 2 h, then washed and cultured with BBI (after). BBI was maintained throughout the culture time period (all). At 48 h post-infection (PI), both HSV-2 genomic DNA and total proteins were extracted from End1/E6E7 cells and subjected to the real-time PCR or Western blot assay.

### 2.6. RNA Extraction and Real-Time PCR

Cellular RNA was extracted from End1/E6E7 cells with Tri-reagent (Molecular Research Center, Cincinnati, OH). Complementary DNA was generated from total RNA using random priming and MMLV reverse transcriptase (Promega Co., Madison, WI, USA). Real-time PCR was performed using SYBR green PCR master mix (Bio-Rad Laboratories, Hercules, CA, USA). All values were normalized to GAPDH mRNA. The oligonucleotide primer sequences are listed in Table 1. 

### 2.7. Western Blot Analysis

End1/E6E7 cells treated with or without BBI were harvested by using RIPA lysis buffer (Beyotime Institute of Biotechnology, Shanghai, China) supplemented with 1% protease inhibitor cocktail (Sigma, MO, USA) and 1% phosphatase inhibitors mixture (Applygen, Beijing, China). The cell lysates were centrifuged at 12,000 × *g* for 15 min, and then the supernatant was collected and quantified by a BCA protein assay kit (Beyotime Institute of Biotechnology). The soluble proteins were separated by SDS-PAGE. After being transferred to a PVDF membrane (BioRad, Hercules, CA, USA), the membrane was blocked by 5% nonfat milk at room temperature for 2 h, followed by incubation with primary antibodies overnight at 4 °C. The PVDF membrane was then washed with TBST and further incubated with horseradish peroxidase-conjugated second antibody. The membranes were washed with TBST, and the immunoblots were developed with enhanced chemiluminescence detection (ECL, Amersham, UK).

### 2.8. Statistical Analysis

Data were shown as the mean ± standard deviation (mean ± SD) and analyzed by Student’s *t*-test using GraphPad Prism for Windows version 5.0 (GraphPad Software, La Jolla, CA, USA), and * *p* < 0.05 was considered as statistically significant results.

## 3. Results

### 3.1. BBI Inhibits HSV-2 Infection of End1/E6E7 Cells

**Figure 1:**
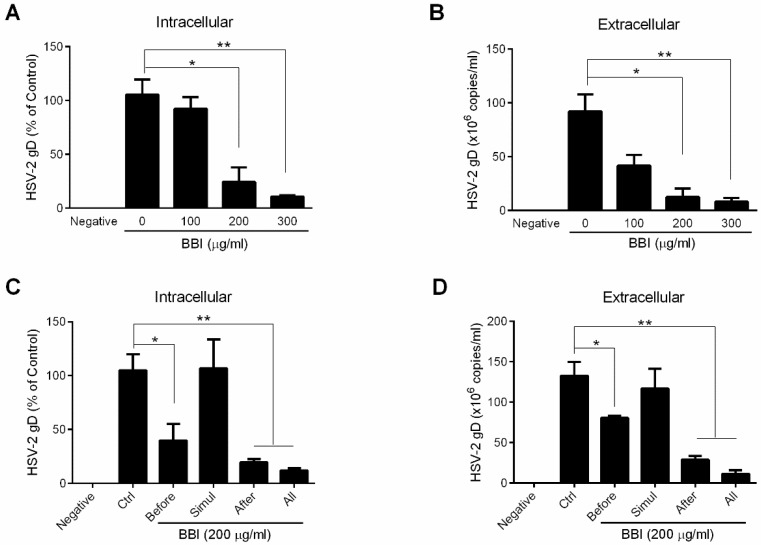

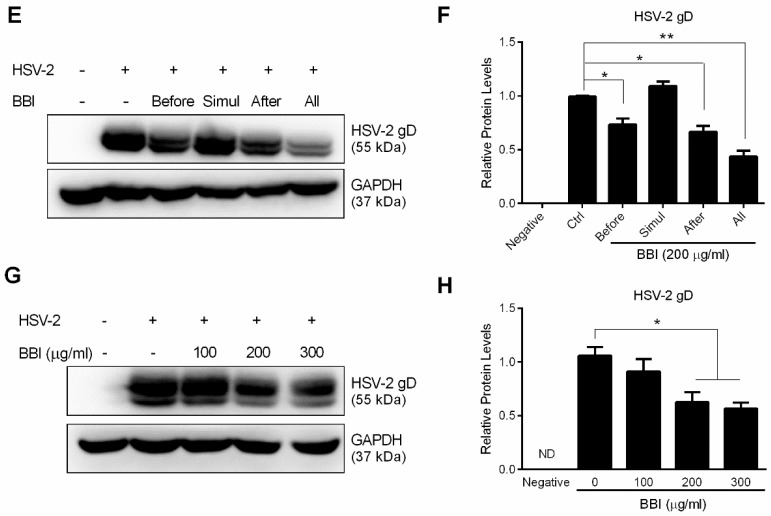
BBI inhibits HSV-2 infection. (**A**,**B**) End1/E6E7 cells were pretreated with BBI at indicated concentrations for 24 h, and then infected with HSV-2 (MOI of 0.001) for 2 h, cells were washed with PBS and maintained with or without BBI for 48 h. Total DNA extracted from (**A**) cells and (**B**) culture supernatant was measured by the real-time PCR using specific HSV-2 gD primers for HSV-2 gD quantification. (**C**–**E**) End1/E6E7 cells were pretreated with BBI (200 μg/mL) for 24 h, then washed with PBS and infected with HSV-2, and then cultured without BBI (before); End1/E6E7 cells were treated with BBI and infected with HSV-2 simultaneously for 2 h, then washed with PBS and cultured without BBI (simul); End1/E6E7 cells were infected with HSV-2 for 2 h, then washed with PBS, cultured with BBI (after); BBI was maintained throughout the cell culture time period (all). At 48 h PI, (**C**) intracellular DNA, (**D**) extracellular DNA, and (**E**) total proteins were collected and analyzed by the real-time PCR or Western blot for HSV-2 gD gene expression. (**G**) End1/E6E7 cells were infected with HSV-2 for 2 h and then treated with BBI at the indicated concentrations, total cellular proteins were collected and subjected to Western blot. (**F**,**H**) Densitometry analysis of the blots shown in E and G was performed with ImageJ 1.44 software. Data shown were obtained as mean ± SD from three independent experiments (* *p* < 0.05, ** *p* < 0.01).

To determine the anti-HSV-2 effect of BBI, End1/E6E7 cells were pretreated with BBI for 24 h and followed by HSV-2 infection. As shown in Figure 1 A,B, BBI-treated cells had lower levels of intracellular and extracellular HSV-2 gD DNA than untreated cells. To further determine the anti-HSV-2 effect of BBI, End1/E6E7 cells were treated with BBI under different treatment conditions (before, simul, after, and all). As shown in Figure 1C–F, although BBI treatment of End1/E6E7 cells during HSV-2 infection (simul) showed little effect on HSV-2 infection, pretreatment of End1/E6E7 cells with BBI (before) or treatment of the cells with BBI after HSV-2 infection (after) significantly inhibited HSV-2 infection at both DNA and protein levels. Treatment of the cells with BBI under all three conditions (all) was the most effective in HSV-2 inhibition (Figure 1C–F). In addition, a dose-dependent antiviral effect was observed in the cells treated with BBI after HSV-2 infection (Figure 1G,H). To determine whether the anti-HSV-2 effect of BBI was due to cytotoxicity, we examined the effect of BBI on the viability of End1/E6E7 cells. As shown in Figure S1, BBI at a concentration as high as 600 μg/mL had little cytotoxicity to End1/E6E7 cells.

### 3.2. BBI Suppresses HSV-2 Gene Expression

**Figure 2:**
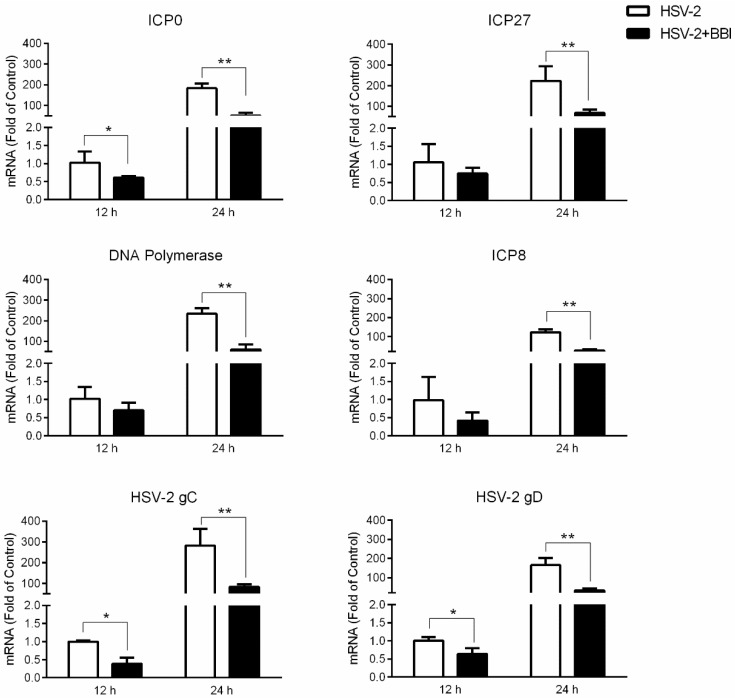
Effect of BBI on HSV-2 gene expression. End1/E6E7 cells were infected with HSV-2 (MOI of 0.002), and then cultured in the presence or absence of BBI (200 μg/mL). Cellular RNAs were extracted from the virus-infected cells at 12 h or 24 h PI, and the expression of HSV-2 *IE*, *E* and *L* genes were analyzed by the real-time PCR. All results were mean ± SD of triplicate cultures, representative of three independent experiments (* *p* < 0.05, ** *p* < 0.01).

To investigate the effect of BBI on HSV-2 genes expression, we examined several viral genes, including two immediate early genes (*IE*, ICP0 and ICP27), two early genes (*E*, ICP8 and DNA polymerase) and two late genes (*L*, HSV-2 gC and gD). As shown in Figure 2, BBI could inhibit the expression of HSV-2 *IE*, *E* and *L* genes in the infected End1/E6E7 cells.

### 3.3. BBI Activates the JAK/STAT Signaling Pathway

**Figure 3:**
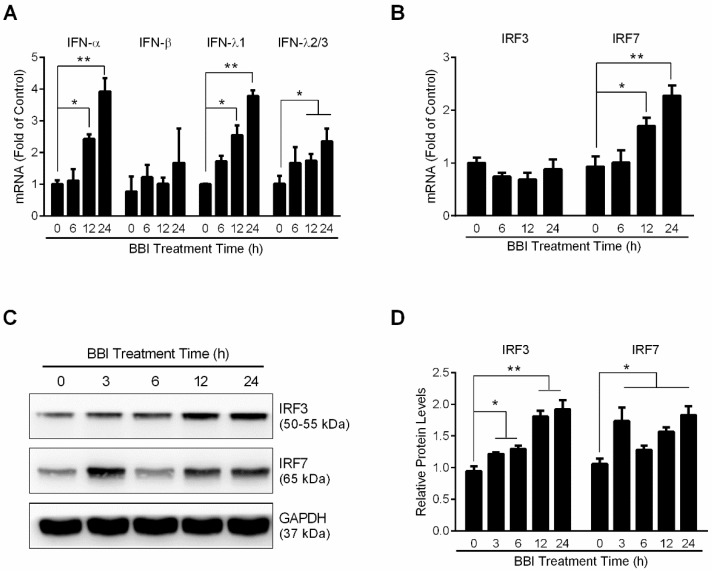
Effect of BBI on IFNs and IRFs expression in End1/E6E7 cells. End1/E6E7 cells were treated with or without 200 μg/mL BBI for indicated times. (**A**,**B**) Cellular RNAs extracted from cells were subjected to the real-time RT-PCR for IFN-α, IFN-β, IFN-λ1, IFN-λ2/3, IRF3, IRF7 and GAPDH mRNA. (**C**) Cellular proteins were collected and subjected to Western blot with the antibodies against IRF3 and IRF7. Representative data from three independent experiments were shown (* *p* < 0.05, ** *p* < 0.01). (**D**) Densitometry analysis of the blots shown in C was performed with ImageJ 1.44 software.

**Figure 4:**
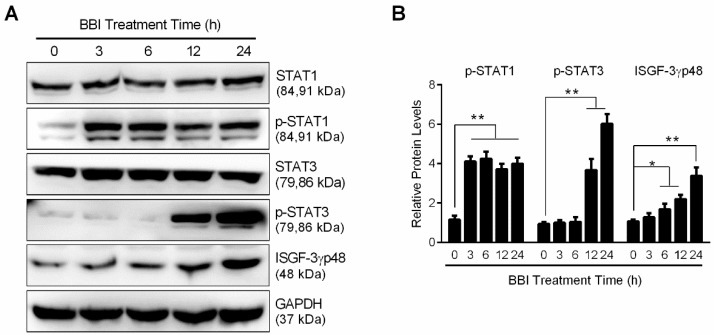

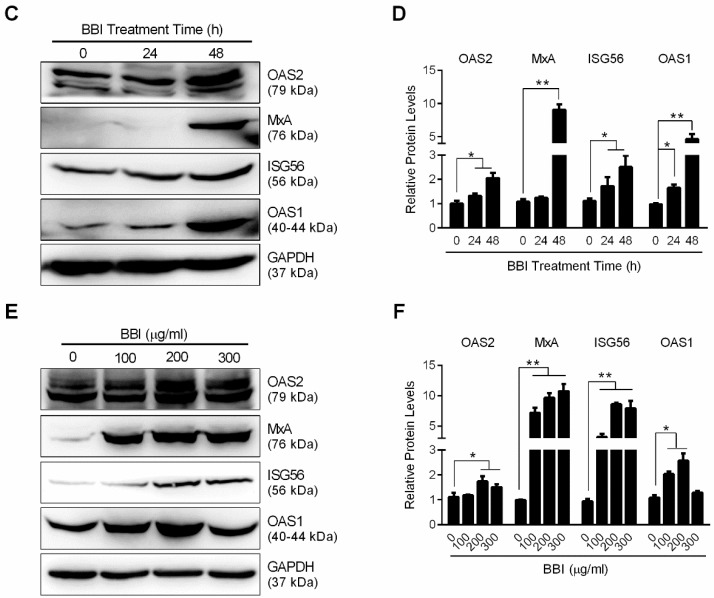
Effect of BBI on the JAK/STAT pathway and ISGs expression. End1/E6E7 cells were treated with or without 200 μg/mL BBI for indicated times. (**A**) Proteins were collected and analyzed with the antibodies against STAT1, p-STAT1, STAT3, p-STAT3, ISGF-3γp48, and GAPDH. (**C**) Proteins were collected and analyzed with the antibodies against OAS2, MxA, ISG56, OAS1, and GAPDH. (**E**) End1/E6E7 cells were treated with BBI at indicated concentrations for 48 h, total proteins were subjected to Western blot. Representative data from three independent experiments were shown. (**B**,**D**,**F**) Densitometry analysis of the blots shown in A, C and E were performed with ImageJ 1.44 software. Data shown were obtained as mean ± SD from three independent experiments (* *p* < 0.05, ** *p* < 0.01).

To further study the mechanisms by which BBI inhibits HSV-2 infection (Figure 1C–F), we examined whether BBI could activate IFN-based immunity in End1/E6E7 cells. As shown in Figure 3A, BBI pretreatment of the cells induced IFN-α, IFN-λ1, and IFN-λ2/3 expression, but showed little effect on IFN-β expression. In addition, BBI induced the expression of IRF3 and IRF7 at the protein level (Figure 3B–D). To determine whether the induction of IFNs is responsible for the activation of JAK/STAT signaling pathway, we analyzed the impact of BBI on the phosphorylation of STAT1 and STAT3. As shown in Figure 4A,B, BBI treatment of the cells induced the expression of p-STAT1, p-STAT3, and ISGF-3γp48 (IRF9). In addition, BBI treatment of End1/E6E7 cells also enhanced the expression of several key antiviral ISGs, including OAS2, MxA, ISG56, and OAS1 (Figure 4C–F).

### 3.4. BBI Inhibits the Cellular UPS

**Figure 5:**
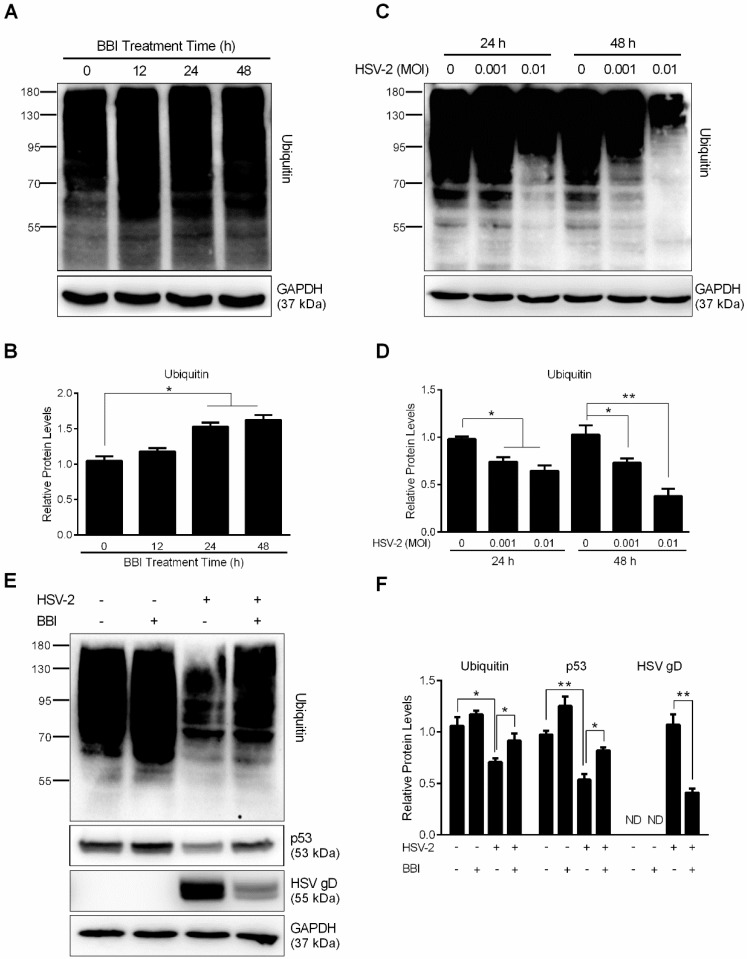
Effect of BBI on HSV-2-induced degradation of ubiquitinated proteins. (**A**) End1/E6E7 cells were treated with BBI for indicated times. (**C**) End1/E6E7 cells were infected with HSV-2 at the indicated MOI, and proteins were collected from the infected cells at 24 h or 48 h PI. (**E**) End1/E6E7 cells were pretreated with or without BBI for 24 h, then infected with HSV-2 (MOI of 0.001) for 2 h, and then cultured with or without BBI. Proteins were subjected to Western blot with the antibodies against ubiquitin, p53, HSV gD or GAPDH. Representative data from three independent experiments were shown. (**B**,**D**,**F**) Densitometry analysis of the blots shown in A, C, and E was performed with ImageJ 1.44 software. Data shown were obtained as mean ± SD from three independent experiments (* *p* < 0.05, ** *p* < 0.01).

The cellular functional ubiquitination plays a significant role in HSV-1 and HSV-2 replication, and the inhibition of cellular ubiquitin‒proteasome system (UPS) could impair HSV infection [28]. As a natural protease inhibitor, it is likely that BBI has an inhibitory effect on cellular ubiquitination. We thus examined whether BBI can inhibit the cellular proteasome-dependent proteolysis. As shown in Figure 5A,B, BBI treatment of End1/E6E7 cells resulted in the accumulation of the cellular ubiquitinated proteins. Further experiments showed that HSV-2 infection facilitated the degradation of ubiquitinated proteins (Figure 5C,D). The treatment of End1/E6E7 cells with BBI resulted in the accumulation of p53 protein. In addition, BBI could reduce HSV-2-mediated downregulation of p53 and loss of ubiquitinated proteins (Figure 5E,F).

### 3.5. BBI Inhibits HSV-2-Induced Activation of NF-κB and MAPK

**Figure 6:**
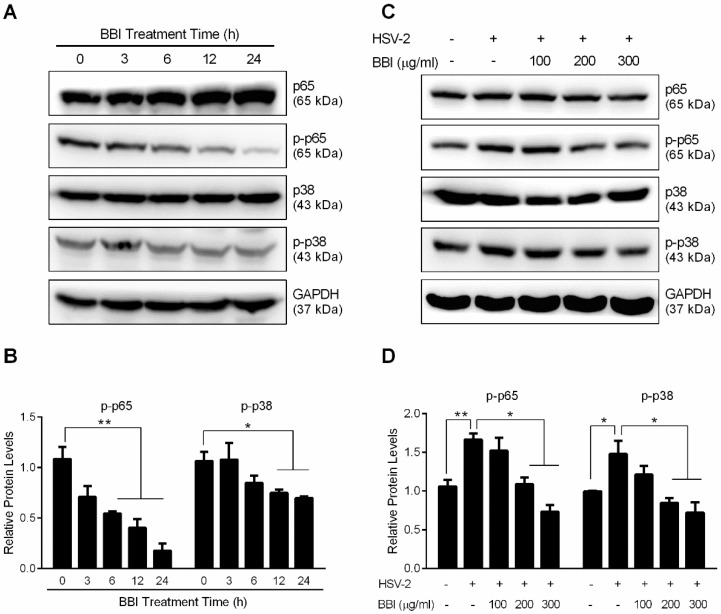
Effect of BBI on HSV-2-induced NF-κB and MAPK activation. (**A**) End1/E6E7 cells were treated with BBI (200 μg/mL) for indicated times. (**C**) End1/E6E7 cells were infected with HSV-2 (MOI of 0.001) for 2 h, then cultured with BBI at indicated concentrations. Cellular proteins were extracted and subjected to Western blot assay. Representative data from three independent experiments were shown. (**B**,**D**) Densitometry analysis of the blots shown in A and C was performed with ImageJ 1.44 software. Data shown were obtained as mean ± SD from three independent experiments (* *p* < 0.05, ** *p* < 0.01).

HSV infection can activate NF-κB and MAPK signaling pathways, which induces inflammation and cell apoptosis, a condition favorable for HSV-2 replication [29,30]. We thus investigated the effect of BBI on the HSV-2-induced NF-κB or MAPK signal activation. The results showed that BBI treatment of uninfected End1/E6E7 cells suppressed p-p65 and p-p38 expression in a time-dependent manner (Figure 6A,B). Further experiments indicated that HSV-2 infection enhanced the expression of p-p65 and p-p38 in End1/E6E7 cells, while the treatment of the cells with BBI effectively blocked HSV-2-induced p-p65 and p-p38 expression (Figure 6C,D).

### 3.6. BBI Suppresses HSV-2-Induced Downregulation of Tight Junction Proteins

**Figure 7:**
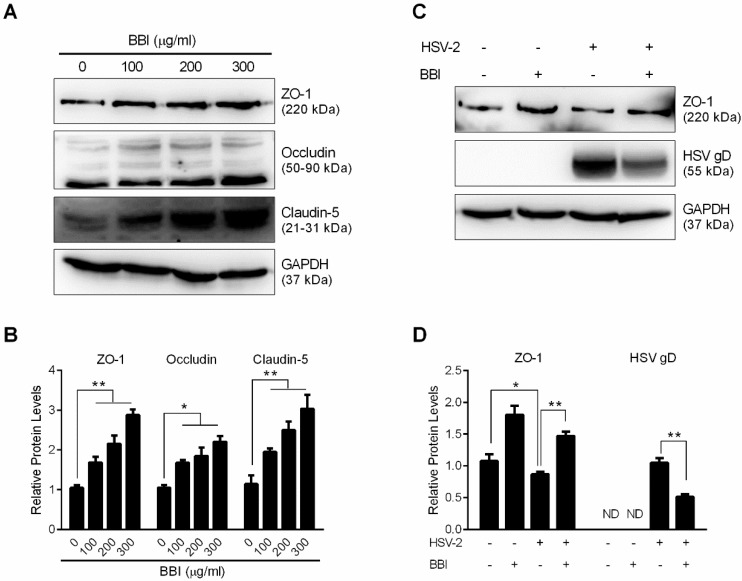
Effect of BBI on HSV-2-induced tight junction protein downregulation. (**A**) End1/E6E7 cells were treated with BBI at indicated concentrations for 48 h. (**C**) End1/E6E7 cells were infected with HSV-2 (MOI of 0.001) for 2 h, then cultured with or without BBI for 48 h. Proteins were collected and subjected to Western blot. Representative data from three independent experiments are shown. (**B**,**D**) Densitometry analysis of the blots shown in A and C was performed with ImageJ 1.44 software. Data shown were obtained as mean ± SD from three independent experiments (* *p* < 0.05, ** *p* < 0.01).

The epithelial cells in female cervical mucosa are crucial in maintaining the junctional integrity and barrier permeability [31]. We thus examined the impact of BBI on the expression of several tight junction proteins (ZO-1, Occludin, and Claudin-5) in End1/E6E7 cells. As demonstrated in Figure 7A,B, BBI treatment of End1/E6E7 cells increased the expression of these tight junction proteins. Further experiments indicated that HSV-2 infection of End1/E6E7 cells selectively decreased ZO-1 expression. BBI, however, could reduce HSV-2-mediated ZO-1 reduction (Figure 7C,D).

## 4. Discussion

Epithelial cells form the first protective barrier in the female reproductive tract, which is vital in preventing viral infections. We [11,32,33] and others [34] have demonstrated that epithelial cells from different organs and regions possess functional TLR3/RIG-I signaling systems, which could be immunologically activated by Poly I:C to trigger the immune responses against viral infections, including HSV-2. In the present study, we demonstrated that BBI could potently inhibit HSV-2 infection of human cervical epithelial cells. The inhibitory effect of BBI was observed under different treatment conditions (before and after HSV-2 infection, Figure 1C–F) and on several viral genes expression (Figure 2). It is known that HSV-2 immediate early (*IE*) genes (ICP0 and ICP27) expression are vital for viral early (*E*) and late (*L*) gene expression, which is a key step in the viral replication [35,36]. HSV DNA polymerase and ICP8 facilitate HSV *L* genes transcription, enhancing viral DNA synthesis and replication [37,38]. HSV gC and gD genes encode viral envelope proteins, the major players in the formation and release of virus particles, as well as promote viral entry [39]. We demonstrated that BBI could inhibit the expression of all these viral genes in End1/E6E7 cells. To inhibit the expression of multiple HSV-2 genes by BBI is highly significant, as it would be difficult for the virus to become resistant or mutant to the BBI-mediated treatment.

Mechanistically, we found that BBI could induce the expression of IFN-α, IFN-λ1 and IFN-λ2/3 expression in End1/E6E7 cells (Figure 3A). Type I IFNs are known to be effective in suppressing HSV infection [40]. Our early study showed that endogenous IFN-λ in TLR3/RIG-I-activated epithelial cells could inhibit HSV-2 infection [11]. The induction of IFNs may be due to the effect of BBI on IFN regulatory factors. We found that BBI treatment significantly enhanced the translational levels of IRF3 and IRF7 (Figure 3B–D), which have key roles in regulating IFNs expression [41,42]. Because the activation of the JAK/STAT signaling pathway plays a crucial role in IFN-mediated innate immune response, we also examined the role of BBI in the activation of the JAK/STAT signaling pathway. In Figure 4A,B, we observed that BBI treatment of End1/E6E7 cells enhanced the expression of p-STAT1, p-STAT3, and ISGF-3γp48. More importantly, BBI induced the expression of several antiviral ISGs, including OAS2, MxA, ISG56 and OAS1 (Figure 4C–F). Some of these ISGs have been shown to have the ability to inhibit HSV-2 infection. For example, the OAS1 protein could directly inhibit HSV-2 proliferation in an RNase L-independent pathway [43]. In addition to HSV-2 infection, the ISGs have been reported to restrict HIV-1 infection as well [44]. However, HSV has evolved multiple strategies to evade host cell-mediated immunity [45,46,47]. Therefore, to induce the ISGs by BBI is beneficial for protecting cervical epithelial cells from HSV-2 infection.

Studies with different cell systems [28,48,49] have shown that Pyrrolidine Dithiocarbamate (PDTC), an antioxidant and an NF-κB inhibitor, could potently inhibit viral infections, including HSV-2, through inhibiting the cellular ubiquitin-proteasome system (UPS). The UPS is a key mechanism for intracellular proteins catabolism, such as degradation of the IκBs and regulation of the NF-κB pathway [50]. We observed that BBI treatment of End1/E6E7 cells increased the expression of cellular ubiquitinated proteins as well as p53, an intracellular tumor suppressor degraded through UPS [51]. The inhibition of UPS by PDTC could suppress the HSV-2-induced IκB-α degradation, may result in inhibition of p65 phosphorylation [28]. Interestingly, studies have reported that p53 is a target of type I IFNs [52] and that p53 could regulate TLR3 expression and function in human epithelial cells [53]. Therefore, it is possible that BBI-mediated induction of IFNs is responsible for the upregulation of p53 protein. In addition to the direct effect of BBI on UPS, we found that BBI treatment of End1/E6E7 cells could block HSV-2 infection-mediated degradation of ubiquitinated proteins and inhibition of p53 (Figure 5E,F). While the precise mechanisms of the BBI action remain to be determined, it is likely that BBI blocks the effect of HSV-2 on ubiquitinated proteins through its unique protease inhibitory function. Studies from different laboratories have shown that NF-κB or MAPK activation contribute to HSV infection in host cells [54,55]. Therefore, to suppress NF-κB or MAPK activation is a therapeutic strategy to inhibit HSV infection [56,57]. We found that while HSV-2 infection activated NF-κB and p38 MAPK signaling pathway, BBI treatment of End1/E6E7 cells suppressed HSV-2-induced NF-κB and p38 MAPK activation (Figure 6). However, the direct effect of BBI on the inhibition of NF-κB activation (p65 phosphorylation) remains to be determined. These observations provide additional mechanisms (blocking the cellular proteasome activity and inhibiting NF-κB and p38 MAPK signaling pathways) for the BBI action on HSV-2 inhibition.

Functional tight junctions are primarily responsible for the integrity and permeability of the epithelial barrier. Disruption of epithelial tight junctions increases the probability of HSV binding to nectin-1, a cellular receptor for HSV entry [58]. HSV infection could damage cervical epithelial cells in human cervical organ culture, and result in the mucosal inflammation, which enhances the susceptibility to HIV-1 infection [59]. We observed that BBI treatment could not only enhance the expression of the tight junction proteins (Figure 7A,B), but also block HSV-2-infection-mediated inhibition of ZO-1 expression (Figure 7C,D).

## 5. Conclusions

**Figure 8:**
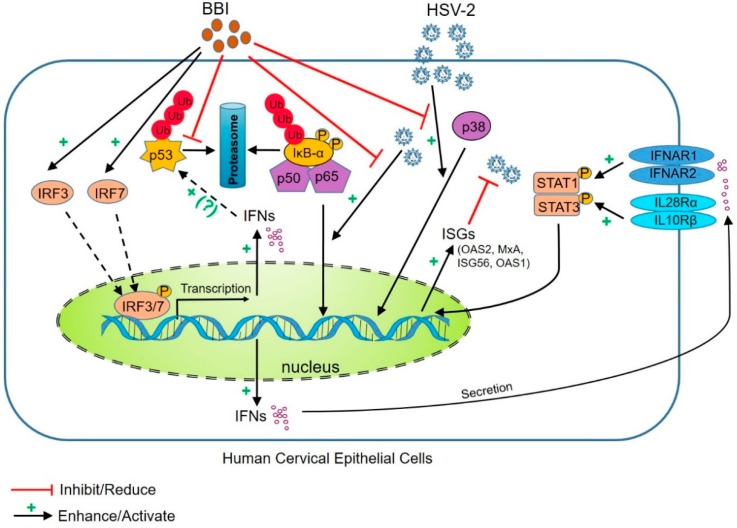
Hypothetical model of the anti-HSV-2 mechanisms of BBI. BBI inhibits HSV-2 infection through the following mechanisms: First, BBI induces IFN-based innate immune activation, resulting in the induction of the antiviral ISGs (OAS2, MxA, ISG56, OAS1) that inhibit HSV-2 replication; Second, BBI inhibits the UPS-mediated degradation of ubiquitinated proteins and p53; Third, BBI inhibits HSV-2-induced NF-κB and p38 MAPK activation.

Taken together, our study for the first time demonstrates that BBI, a protease inhibitor extracted from soybean, could effectively inhibit HSV-2 infection of human cervical epithelial cells through the following mechanisms: 1, induction of the expression of intracellular antiviral factors; 2, suppression of the cellular UPS; 3, inhibition of the HSV-2-induced NF-κB and p38 MAPK activation (Figure 8). In addition, BBI could upregulate the expression of tight junction proteins and block HSV-2 infection-mediated reduction of the ZO-1 protein. These observations, in conjunction with our early studies [25,26], indicate that BBI may have therapeutic potential as a natural and cost-effective agent for the prevention and treatment of HSV-2 and other STIs. However, future in vivo studies with suitable animal models are needed in order to validate these in vitro findings and determine the protective effect of BBI on HSV-2 sexual infection.

## Figures and Tables

**Table 1:** Primer pairs.

Gene Name	Sequence	
	Forward (5′-3′)	Reverse (5′-3′)
GAPDH	GGTGGTCTCCTCTGACTTCAACA	GTTGCTGTAGCCAAATTCGTTGT
IFN-α	TTTCTCCTGCCTGAAGAACAG	GCTCATGATTTCTGCTCTGACA
IFN-β	GCCGCATTGACCATCTATGAGA	GAGATCTTCAGTTTCGGAGGTAAC
IFN-λ1	CTTCCAAGCCCACCCCAACT	GGCCTCCAGGACCTTCAGC
IFN-λ2/3	TTTAAGAGGGCCAAAGATGC	TGGGCTGAGGCTGGATACAG
IRF3	ACCAGCCGTGGACCAAGAG	TACCAAGGCCCTGAGGCAC
IRF7	TGGTCCTGGTGAAGCTGGAA	GATGTCGTCATAGAGGCTGTTGG
HSV-2 ICP0	GTGCATGAAGACCTGGATTCC	GGTCACGCCCACTATCAGGTA
HSV-2 ICP27	TTCTGCGATCCATATCCGAGC	AAACGGCATCCCGCCAAA
HSV-2 ICP8	AGGACATAGAGACCATCGCGTTCA	TGGCCAGTTCGCTCACGTTATT
HSV-2 gC	AAATCCGATGCCGGTTTCCCAA	TTACCATCACCTCCTCTAAGCTAGGC
HSV-2 gD	ATCCGAACGCAGCCCCGC	TCTCCGTCCAGTCGTTTAT
HSV-2 DNA polymerase	GCTCGAGTGCGAAAAAACGTTC	CGGGGCGCTCGGCTAAC

## Supplementary Materials

The following are available online at http://www.mdpi.com/1999-4915/10/10/557/s1.
