# Supplementary material for: Bowman‒Birk Inhibitor Suppresses Herpes Simplex Virus Type 2 Infection of Human Cervical Epithelial Cells
Source: Viruses. 2018 Oct 12;10(10):557. doi: 10.3390/v10100557 (PMC6213026; doi:10.3390/v10100557)
Supplement: Supplementary file 1 [file viruses-10-00557-s001.pdf]

Supplementary Materials

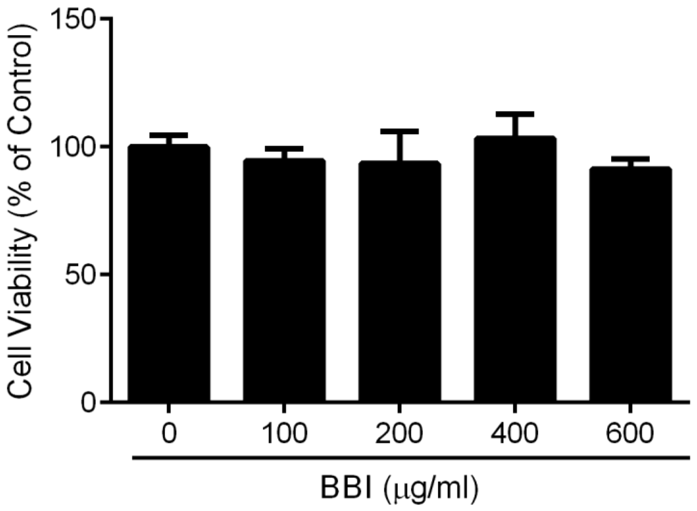

Figure S1. The cytotoxic effect of BBI on End1/E6E7 cells. End1/E6E7 cells were placed in a 96-well plate and cultured in the presence of BBI at the indicated concentrations for 96 h. Cell viability was measured by MTT assay. Data shown were the mean ± SD from three independent experiments.
